# Snakes, Snakes

**Published:** 1887-07

**Authors:** 


### SNAKES! SNAKES!!

In the *Eclectic Medical Journal* (Scudder's), of May, 1887, we read the following:

"ECHIUACEA AUGUSTIFOLIA.—* * * Dr. Meyer claims that this root is a 'blood-purifier,' an antiseptic for internal and external use, superior to any now known to the medical world, and that he has successfully administered it in disorders ot the stomach—cholera infantum, cholera morbus, intermittent, remittent, congestive, and typhoid fevers, spasmodic affections, small-pox, measles, boils, carbuncles, ulcerated sore throat, ulcers of the extremities, etc. In malarial troubles it has no superior. In six cases of typhoid fever, two of the patients were out of bed on the eighth day,

* American Journal of Otalgia, vol. ii, p. 301; 1880.

three on the tenth, and one on the twelfth. Twenty-five drops of the pure tincture injected into the rectum in cases of hemorrhoids, repeating the injections three times per day, will promptly effect a cure. The medicine is also prompt and efficacious in stings from bees, wasps, etc., as well as in poisoning by contact with certain vegetables—one or two doses effecting recovery.

"In six hundred and thirteen cases of rattlesnake bites, with men and animals, pro[illegible]pt cures have been made. Dr. Meyer states: 'I injected some of the rattlesnake poison into my system, on the first finger of the left hand; the swelling was rapid, and in six hours was up to the elbow. At this time I took a dose of the medicine, washed the swelling with it, and laid down to sleep. I slept four hours, and on rising did not find a single sign of swelling on my finger or arm.' The recoveries from rattlesnake bites, under its action, are effected in from two to twelve hours. From his knowledge of its influence upon poisons in the system, Dr. Meyer thinks this agent may prove serviceable in hydrophobia. Last December (26th) he furnished some of it to Prof. I. J. M. Goss, M. D., of Marietta, Ga., who used it in two cases where the parties had been bitten by a rabid dog. About the 12th of March, 1887, he received a line from Professor Goss stating that no indications of hydrophobia with these patients had yet presented."

Here we are called upon, as medical readers, to perform an impossibility—to accept the *ipse dixit* of a mo[illegible]t wonderful doctor—a magician curer—a discoverer of a panacea, the administration of which, in a dose not exceeding 25 minims, will cure, in less time than it takes to read the above, all disorders of the stomach, cholera infantum, cholera morbus, intermittent, remittent, congestive and typhoid fevers, spasmodic affections, small-pox, measles, ulcerated sore throat, ulcers of the extremities, malarial troubles, hemorrhoids, stings of bees and wasps, vegetable poisons, and, last but not least, snake bites! *Six hundred and thirteen* cases of *rattlesnake* bites (mind, *rattlesnake* alone) have been cured by one man! And this is but the singling out of a particular variety of bites from a vast number of venomous stings! If you add thereto the number of bites from cobras, mocassins, cotton-mouths, spreading adders *et id omne genus*, which Dr. Meyer must have cured, but did not deem expedient to mention and to subjoin to the above, you will have ample material to compose a most interesting, or, rather, startling *snake story!*

But the culmination of our amazement was reached when we read that this great Dr. Meyer went so far as to almost immolate himself on the altar of Eclecticism by injecting some of the rattlesnake poison into his own system! At this point we fell into a syncope and precipitately called for medical assistance. We are happy to state that we have somewhat recovered from our shock.

This is an age of skeptics and of critics. People to-day will not even believe that an Eclectic doctor is infallible.

In conclusion, we will say that we can't believe *that* snake story; we doubt that self-inoculation on the part of a sane man of most violent and deadly poison, for the fool-hardy purpose of testing, at his own peril, the value of a *supposed*, *untried* and *unknown* antidote. We do not even propose to put any credence in that report concerning the hydrophobia cases of Prof. I. J. M. Goss, M.D. of Marietta, Ga. We live within speaking and hearing distance of the famous "Gossworks." The latter invariably explode whenever an Eclectic discovery is made; they have been silent for a long while, and the two hydrophobia cases are yet to be heard from—at least in this section! If their history be true, another earthquake must be expected at any moment. E. v. G., M. D.

P. S. By-the-way, the above playful allusion to Professor Goss is intended in no personal or offensive sense, but rather to indicate the Professor's well known and extraordinary enthusiasm in matters Eclectic.

